# “People who have money feed formula to their infants”: a qualitative study of exclusive breastfeeding barriers and potential interventions in Lao People’s Democratic Republic

**DOI:** 10.1186/s12889-026-27416-y

**Published:** 2026-04-23

**Authors:** Souliviengkham Sonephet, Rebecca Reno, Günther Fink, Sengchanh Kounnavong, Jordyn T. Wallenborn

**Affiliations:** 1 Lao Tropical and Public Health Institute, Vientiane, Lao PDR; 2 University of California, Berkeley, CA USA; 3 Swiss Tropical and Public Health Institute, Department of Epidemiology and Public Health, Basel, Switzerland; 4 University of Basel, Basel, Switzerland

**Keywords:** Exclusive Breastfeeding, Mothers, Child under Age 2 Years, Vientiane Capital, Lao PDR

## Abstract

**Introduction:**

The World Health Organization recommends exclusive breastfeeding for the first six months of life as the optimal way of feeding infants. Breastfeeding rates vary widely by geographic region, sociocultural, economic and health system factors. In urban settings such as Vientiane Capital, Lao People’s Democratic Republic, mothers face several barriers that make exclusive breastfeeding challenging. We did a qualitative study to explore these factors and understand the views of mothers, families and health workers on exclusive breastfeeding.

**Methods:**

A qualitative study was conducted in Vientiane Province, Lao People’s Democratic Republic, involving 23 mothers and 21 fathers in focus group discussions, along with in-depth interviews with 4 healthcare workers, 4 Lao Women’s Union members, 4 village health community members, and 4 elders/grandmothers. Data were transcribed, translated, coded, and analyzed using qualitative content analysis.

**Results:**

Participants identified multiple barriers to breastfeeding, including mode of delivery, perceived convenience of formula feeding, maternal health concerns, employment-related challenges, and insufficient milk supply. Limited and inconsistent communication from healthcare providers further contributed to these challenges. Participants also discussed potential interventions to better support breastfeeding practices.

**Conclusion:**

Most participants valued breastfeeding, and most mothers expressed a strong intention to breastfeed exclusively. Strengthening supportive policies, improving breastfeeding education, and fostering breastfeeding-friendly environments may substantially improve exclusive breastfeeding practices, thereby promoting the health and well-being of both mothers and infants.

**Supplementary Information:**

The online version contains supplementary material available at 10.1186/s12889-026-27416-y.

## Background

Human milk is the gold standard of infant feeding. Uniquely composed of bioactive and nutrient factors, human milk promotes healthy development of the infant’s immune system and protects the infant against many common childhood illnesses [1]. Both exclusive and complementary breastfeeding have been shown to reduce child under five mortality preventing an estimated 823,000 deaths per year [2]. Human milk also promotes a healthy life course by supporting brain development and neurocognitive functioning [3].

The World Health Organization advises exclusive breastfeeding for the first six months of life, followed by continued breastfeeding up to two years of age or beyond, alongside appropriate complementary foods [4, 5]. Support of WHO breastfeeding recommendations is essential for countries wanting to achieve the United Nations sustainable development goals by reducing child malnutrition, preventing avoidable childhood illness, and improving child survival [2].

There are marked differences in breastfeeding rates between developed and developing countries, where increasing the proportion of women exclusively breastfeeding until 6 months could potentially have a large impact on economic and health progress [6]. In Lao PDR, only 44.9% of children aged 0–5 months were exclusively breastfed in 2017. In Vientiane, the capital of Lao PDR, less than a quarter (21.0%) of mothers exclusively breastfeed and less than half (42.3%) are predominantly breastfed between 0 and 5 months [7].

Overcoming the low breastfeeding rates in Lao PDR has proven difficult. A collaborative effort by the Lao PDR Ministry of Health, the European Union and UNICEF implemented an intervention to encourage breastfeeding education and support among health workers, mothers and families [8, 9]. However, this intervention was unsuccessful – likely being impacted by differing breastfeeding practices in urban-rural communities. A recent study found that rural mothers are more likely to breastfeed compared to urban mothers, these differences in breastfeeding practices were associated with educational level, wealth index, urban–rural residence, and broader socioeconomic structures [9].

Identifying effective interventions to increase exclusive and complementary breastfeeding rates in Lao PDR is a priority for the country. In 2021–2025 The Lao Ministry of Health launched a national breastfeeding campaign to increase the rate of exclusive breastfeeding in all provinces, including Vientiane capital city, from 45% to 60% by 2025 [10].

This study aims to explore barriers to exclusive and continued breastfeeding in Vientiane, Lao PDR. Secondary objectives include examining breastfeeding-related norms, household decision-making processes, and community support structures as contextual factors shaping breastfeeding practices and duration. The findings are intended to inform future breastfeeding and human milk research in urban Lao settings.

## Methods/design

### Participants and recruitment

We conducted a semi-structured, exploratory, qualitative study, in four Vientiane districts: Chanthabuly, Sikhottabong, Sangthong and Pagum Pakgnum. Focus groups and key informant interviews were conducted among five separate groups:

1. Focus groups with 23 women with young children.
2. Focus groups with 21 men with young children.
3. Key informant interviews with 4 representatives from the Ministry of Health.
4. Key informant interviews with 4 healthcare workers (i.e. traditional birth attendants, midwives).
5. Key informant interviews with 4 respected elders, including teachers.

**Fig. 1:**
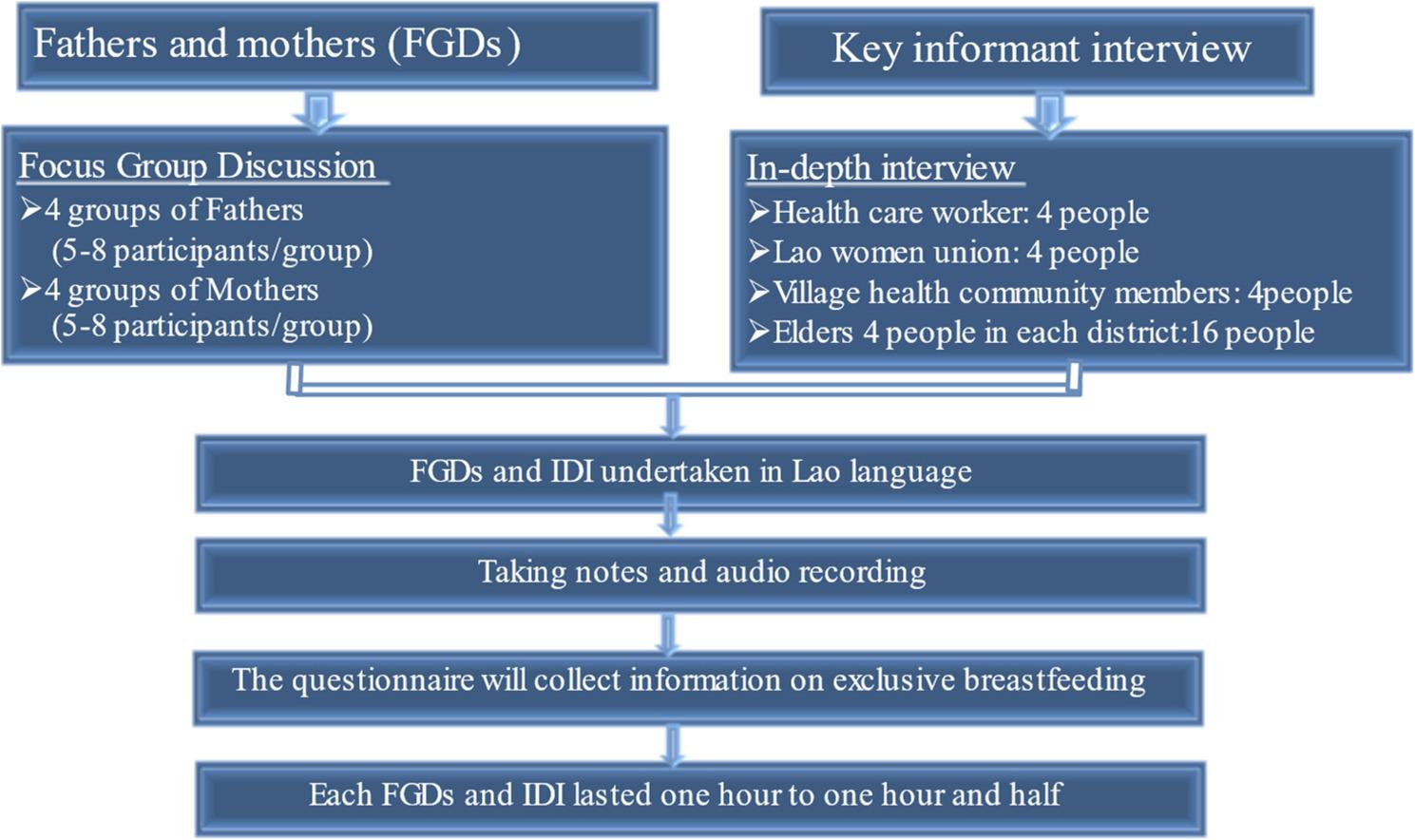
Sampling procedure and data collection for qualitative study in Vientiane, Lao PDR

Focus group discussions were conducted separately with mothers and fathers across the four districts, with 5–8 participants per group, and data collection continued until thematic saturation was reached. Figure 1 illustrates the sampling procedure and data collection process.

### Eligibility criteria

*Women and men* were eligible if they had: (1) at least one child under age 2 years, (2) lived in Vientiane in one of the following districts: Chanthabuly, Sikhottabong, Sangthong, or Pakgum Pakgnum, (3) did not have a medical, intellectual, or psychological disability, and (4) agreed to participate and sign an informed consent. If the participant was under 18 years of age, written informed consent was obtained from both the participant and a parent or legal guardian, in accordance with national ethical guidelines and the approved study protocol.

*Village Leaders* were eligible if they (1) lived in Vientiane in one of the following districts: Chanthabuly, Sikhottabong, Sangthong, or Parkngum, (2) did not have a medical, intellectual, or psychological disability, and (3) agreed to participate and sign an informed consent.

*Ministry of Health and Healthcare Workers* were eligible if they, (1) did not have a medical, intellectual, or psychological disability, (2) had professional experience in maternal and child health services, (3) agreed to participate and sign informed consent.

### Study recruitment

Key individuals within predefined groups were identified by village leaders in each district. These individuals then nominated potential participants, who were approached by trained research staff from the Lao Tropical and Public Health Institute to gauge their interest in participation. Interested individuals, upon signing a consent form, received a reminder card with focus group details. To enhance participation, confirmation letters were provided to each group. Key informants, identified individually by village leaders, were also interviewed.

### Procedure

The study adhered to a protocol for focus groups and interviews. The team introduced themselves, stated study objectives, obtained consent, and facilitated discussions on exclusive breastfeeding. Participants received snacks and soap as small gifts. An Android-based tablet computer was used to record participant information and complete study forms using the Open Data Kit (ODK) platform. Focus group discussions and interviews were audio-recorded using a separate digital voice recorder and later transcribed for analysis.

### Trustworthiness

Established qualitative research strategies were used to ensure methodological rigor. Credibility was enhanced through triangulation of data sources, including focus group discussions and key informant interviews across multiple participant groups. All interviews and focus group discussions were transcribed and independently coded by members of the research team, with discrepancies discussed and resolved through consensus. Dependability was supported using a standardized interview guide and a consistent data collection protocol across all study sites. Confirmability was strengthened through regular analytic discussions within the research team, including supervisory review and feedback on data interpretation from a senior researcher at the Lao Tropical and Public Health Institute, Swiss Tropical and Public Health Institute, as well as validation of interpretations with Lao members of the research team to ensure cultural and contextual accuracy.

Data collection continued until thematic saturation was reached, defined as the point at which no new themes emerged from additional interviews or focus group discussions. Saturation was assessed across participant groups to ensure that diverse perspectives relevant to the study objectives were adequately captured. Participants included mothers, fathers, healthcare workers, elders, and representatives from the Ministry of Health in different districts, allowing comparison across gender and roles. The final number of interviews and focus group discussions was therefore considered sufficient to support the aims of this exploratory qualitative study.

### Data analysis

A qualitative content analysis approach was used to analyze the data; codebook was co-created by members of the research team using an inductive approach. All transcripts were dual coded separately, then compared. Inter-rater reliability was assessed using a pooled Cohen’s kappa coefficient [11]. Data from different participant groups were initially coded separately to capture group-specific perspectives. During subsequent analytic stages, codes were compared and synthesized across groups to identify shared themes related to breastfeeding barriers, while noting relevant differences in perspectives across groups. The analysts met on a standing bi-monthly basis to discuss progress and resolve discrepancies. Discrepancies were resolved through consensus, or through validation with a Laotian member of the research team.

## Results

The following results discuss major barriers to breastfeeding (Table 1).

**Table 1:** Number of coded barriers to breastfeeding by participant type

Barriers to Breastfeeding	Mothers (*N*)	Fathers (*N*)	Lao Women’s Union (*N*)	Healthcare workers (*N*)	Health Community Leaders (*N*)	Grandmothers (*N*)	Elders (*N*)	Total coded references (*N*)
Insufficient breast milk	30	14	11	4	1	0	8	68
Employment barriers	16	10	15	7	7	2	7	64
Maternal health	5	3	9	5	5	0	2	29
Formula feeding convenience	1	1	5	1	0	0	1	9
Caesarean delivery	3	1	1	0	0	0	3	8
Concerns about breast appearance	4	0	1	0	0	0	1	6
Breastfeeding challenges	0	0	0	4	0	0	2	6

“Coded references” represent the number of qualitative data segments coded for each theme across transcripts. These counts indicate the frequency with which themes appeared in the dataset and should not be interpreted as measures of prevalence or magnitude of effect

### Insufficient milk supply

The primary reason for discontinuing breastfeeding was insufficient milk supply, often occurring immediately after delivery. Some participants mentioned a lack of knowledge on initiating milk production or milk letdown. Healthcare workers noted that perceptions of insufficient milk supply postpartum often resulted in formula supplementation. Several participants linked early perceptions of insufficient milk supply to experiences around delivery and the immediate postnatal period, particularly among mothers who delivered by caesarean section. Delayed initiation of breastfeeding, postoperative pain, and limited practical breastfeeding support immediately after birth were described as contributing to early formula supplementation.

*"Some people say that breast milk takes time to come in enough, the baby cries a lot, or the milk is not enough to satisfy the baby’s hunger, so they supplement with formula."*

Others described how mothers experienced insufficient milk supply as the baby grew, occurring between 1- and 9 months postpartum. One father described this:

*“Breastfeeding is a problem because my children are not full. When they get breastfeed one to two hours they start crying because it’s not full*,* we have to combine a milk powder. We always talk about this problem and always have a problem everyday about [how to] raise the children”.*

Participants across multiple groups, including mothers, Lao Women’s Union members, and healthcare workers identified multiple contributing factors to insufficient milk supply, including genetics, maternal age (i.e. older women), mode of delivery (i.e. caesarean section), and returning to work with an insufficient breast pump or lack of time/space to express breast milk. One participant from the Lao Women Union in Chanthabuly suggested that insufficient milk supply is a more recent phenomenon:

…*breast milk is not enough currently. In the past*,* the mother feeds only breast milk*,* but now it is not possible*.

Some cultural postpartum practices were also believed to impact milk production. One healthcare worker in Sikhottabong explained:

*"After discharge from the hospital*,* in Lao tradition they believe that staying on a hot bed [yoo fai] or lying by the fire after childbirth [Yoo kam] causes no breast milk to feed the baby."*

Insufficient food for the mother, food type including the consumption of “forbidden food/drink that should not be eaten after giving birth” was also hypothesized to influence milk supply. Some expressed that warm water could increase milk production, and conversely that drinking cold water can contribute to insufficient milk supply.

*"Cold water is the reason that breast milk [did] not come."*

### Employment status

Returning to work was a significant factor affecting breastfeeding duration, impacting both milk production and time commitment. Participants indicated that a mother’s return to work directly influenced the decision to cease breastfeeding entirely or supplement with formula or cow’s milk. This was evident among those who had not returned to work and mothers planning to stop breastfeeding upon their return to work.

This connection between postpartum return to work and decreased breastfeeding duration was the only theme mentioned across all participant groups. One interviewee from the Lao Women Union in Chanthabuly spoke about the economic factors driving the return to work, and how that shapes infant feeding practices.

*"This is not culture; this is depending on the convenience of each person because someone cannot breastfeed when women must help the family earn money. If breastfeeding only, it is not possible for this. Someone will pump breast milk and provide a bottle to feed the infant because they do not want to be absent from work. But this is only in a family that does not have enough money. If (they have money) most people will give formula milk."*

The timeframe for mothers’ returns to work ranged from one month to six months postpartum. One participant described how an early return to work interrupted breastfeeding practices.

*"Mothers that are not staff of the government have a short duration maternity leave. It means they will (not) have enough duration for breastfeeding as recommended. They also don’t have time to hand pump the breast milk and keep it in the refrigerator."*

The connection between returning to work and lower breastfeeding duration was particularly the case for women who worked outside of their village or far from home.

*"Most of them are laborers, some families are very poor, and sometimes they give birth less than 5 or 6 months (ago). They must go to work to earn money…We advise them to squeeze breast milk into the bottle, but in case they work very far from home (and) cannot come back home, they will give them the formula milk."*

As with insufficient milk supply, participants, particularly elders, discussed how working and breastfeeding have changed over time:

*When my daughter gave birth, I had suggested her to breastfeed first. Breastfeeding before she went to work and after I would take care of her baby at home until she finished working…in the past someday we went to work and, in the afternoon, we had a break time so we could come back home to breastfeed our baby, then we would go back to work after finished breastfeeding. Today, we go to work in the morning until 8pm. So many difficult things (in today’s society).*

### Maternal health

Another factor for early breastfeeding cessation identified across multiple participant types was the mother’s health and/or the infants exposure to disease through the mother’s milk. Some participants also perceived that limited breastfeeding counselling and follow-up during the postnatal period reduced mothers’ confidence in continuing exclusive breastfeeding, particularly following surgical delivery. Poor maternal health was cited as a general factor impeding breastfeeding. Several participants emphasized the importance of diet, sufficient rest and good health for the mother. If the mother was not in good health, she may experience insufficient milk supply resulting in infant malnourishment and a weak immune system.

*The impact of breastfeeding depends significantly on a woman’s health. If a mother is undernourished or lacks proper nutrition, breastfeeding may not yield the desired benefits. However, when a mother is in good health, well-fed, and not financially constrained, she can provide high-quality breast milk to her child. I recall observing a woman near my house who was thin, yet her child thrived and was remarkably healthy due to the nourishing breast milk she provided.*

Additionally, participants indicated that disease (including chronic disease) and/or infection may also inhibit breastfeeding:

*[The] Mothers with chronic disease*,* if they breastfeed a baby*,* maybe the disease comes through the milk.*

Mothers’ health and breast milk was said to be negatively impacted by giving birth via caesarean section. One participant from the Lao Women Union in Chanthabuly shared her perception of how a surgical birth impacts breastfeeding long term:

*I think that changing of birth delivery is the same. may I cross to explain about a delivery*,* most people that operated will have not good health as natural delivery. their health is weak and not healthy. After delivery*,* they will breastfeed for 3 months*,* 6 months and then they will reduce to feed.*

### Convenience of formula

Participants described this convenience in relation to flexibility for working mothers, reduced physical burden, ease of feeding by other caregivers, and greater mobility outside the home. These perceptions were especially common among younger mothers, first-time mothers, and families with higher socioeconomic status.

“*One thing depends on the social status. People who have money feed formula milk to their infant. They should not give formula milk but should breastfeed. Presently someone who has enough money will buy milk to feed their infant. It is a modern society. They want to be easy and comfortable in life. They can do everything and go everywhere if feeding formula milk.’’*.

The convenience of formula feeding was identified by one father as it allowed the mother to sleep, as opposed to waking up frequently during the night to breastfeed. Finally, the complexities of expressing breastmilk for working mothers was juxtaposed with the ease of supplementation or formula feeding.

### Mode of delivery

Surgical birth (i.e., caesarean section), lack of health education and practice to support exclusive breastfeeding from healthcare workers, including healthcare service was expressed as a barrier to breastfeeding initiation and duration caused by insufficient milk supply and long-term effects on the mother’s health, as mentioned above. Several mothers described that separation from their infant and limited breastfeeding assistance during the immediate postnatal period following caesarean section contributed to early supplementation, participants also discussed the ways that a caesarean section impacted breastfeeding. For some, this occurred immediately after birth. One mother from Pakgnum explained.

…*because baby cry*,* when I gave birth I still in operation room and my baby always cry. So*,* my husband bought powder milk for the baby*,* and they quiet suddenly after they got milk bottle.*

Two other mothers discussed supplementation during the postpartum period, as breastfeeding was difficult and painful due to the surgical wound.

### Breastfeeding challenges

Healthcare workers noted that, beyond insufficient milk supply, maternal physiological challenges like hollow or inverted nipples and plugged ducts negatively affect optimal breastfeeding initiation or continuation. Despite antenatal counselling, healthcare workers acknowledged that practical breastfeeding support in the postnatal period was often constrained, which in some cases resulted in early formula use. In Sangthong, one healthcare worker described how this led to supplementation or a full transition to formula feeding.

*We start to promote health during Antenatal Care. During pregnancy examinations we also give them recommendations about how to exclusively breastfeed and how to adjust when the mother has a problem with breastfeeding. Sometimes if the mother cannot breastfeed because of hollow nipples*,* we recommend powdered milk because the powdered milk company advertise with us in the department*,* such as lactogen brand.*

Other identified challenges related to breastfeeding were focused on the infant’s unwillingness or inability to nurse.

It is important to note that while these factors are presented independently in the analysis, in reality they are not distinct. In multiple instances, participants identified intersections between the aforementioned factors that shaped the capacity of a woman to breastfeed her infant for the optimal duration:

*The truth - I think that women who breastfeed have to take a rest and [receive] help from their families. Women should have enough food to breastfeed infants to have good health. If the mother is the only one who works*,* they do not have rest time. After three months the mother has to go back to work*,* then the time is not enough. The infants do not have enough breast milk. Then it is necessary to (1) give formula milk and (2) give food to the infant.*

While these barriers are presented as distinct themes, there is also significant overlap and multiple interconnections between them.

Potential Interventions to Promote Breastfeeding: Participants, when discussing interventions to overcome breastfeeding barriers, highlighted the potential of cash transfers or incentives. While most fathers, mothers, and health workers disagreed with cash transfers, they favored non-monetary incentives like gifts or toys for children and supportive policies from the health department.

*It’s not reasonable, to give money that like hiring them to breastfeed, in fact, that should depend on themselves.*

*-father in Pakgnum district*

*If we give money to them, they will follow us temporarily specifically when we have money to support them. If no money they will stop also.*

*- healthcare worker in Sikhottabong district*

*I would like to suggest that the office increase the number of days off for postpartum mothers and implement a policy that allows mothers to stay at home while still receiving their salary.*

*- mother in Chanthabuly district*.

Furthermore, some elders and village leaders agreed with payment but also recognized its potential problems. The participants also suggested advertising and community activities to promote breastfeeding.

*Ohh! It would be beneficial if the office could implement a policy that allows postpartum mothers to stay at home and take care of their babies while still receiving their salary. This would enable mothers to focus on their newborns without worrying about financial constraints. A six-month support period would be ideal for this purpose, that sounds great.*

*-grandmother in Sikhottabong district*

*If no one pays they will not do that, the best way is the mother should earn money by themself.*

*-grandmother in Sang thong district*

*Teenagers nowadays may not follow or believe recommendations from family or parents. It would be beneficial to have more advertising from health workers to raise awareness about health issues among teenagers.*

*-grandmother in Chanthabuly district*.

## Discussion

Our study in Vientiane, Lao PDR, identified barriers to breastfeeding. Early cessation of breastfeeding was attributed to insufficient milk supply, employment status, maternal health, delivery mode, convenience of formula, and general breastfeeding challenges. Across these themes, participants’ accounts highlight gaps in breastfeeding support across the continuum of care, including antenatal counselling, delivery practices, and postnatal follow-up. While breastfeeding information was reportedly provided during antenatal care, limited hands-on support during delivery and immediately after birth, particularly following caesarean section contributed to delayed initiation and early supplementation. Proposed interventions varied between groups; cash incentives were endorsed by key informants while mothers and fathers preferred social media campaigns or increased education.

Identified barriers to breastfeeding are consistent with findings from other geographic settings. In Northwest Ethiopia, suboptimal breastfeeding practices, including delayed initiation and early feeding challenges, have been reported [12]. While this comparison provides useful conceptual insight into structural barriers to breastfeeding, greater emphasis is placed on evidence from Southeast and East Asian contexts that are more comparable to Lao PDR. The countries in this region share similar sociocultural norms, health system structures and exposure to breastmilk substitute marketing. In Thailand, the convenience of formula feeding and alternative infant feeding practices have been identified as important barriers to breastfeeding [13]. Similarly in Vietnam, studies have documented suboptimal knowledge, attitudes, and practices related to exclusive breastfeeding, including early introduction of breastmilk substitutes [14]. Exposure to formula milk marketing has also been shown to negatively influence exclusive breastfeeding practices in Lao PDR [15]. Some participant quotations also suggested potential exposure to breastmilk substitute promotion within health facilities. However, systematic assessment of marketing practices was beyond the scope of this qualitative study. Although this study did not directly assess marketing practices, participants’ descriptions of formula as a convenient and socially acceptable feeding option likely reflect broader commercial and social influences documented in previous research. Mothers further encounter health-related challenges compounded by insufficient support from health services, particularly in the postpartum period following cesarean section deliveries, as reported in studies from China and Malaysia [16, 17]. Addressing these multifaceted barriers requires strengthening health service support, improving breastfeeding education, and providing tailored assistance to mothers experiencing breastfeeding difficulties [18]. Participants described ongoing efforts to promote breastfeeding, including antenatal education, advice from healthcare workers, community outreach activities, and support from women’s unions and elders. However, these efforts were often perceived as insufficient to overcome structural barriers such as short maternity leave, limited workplace support, and inadequate postnatal follow-up, particularly in urban settings.

Cash transfers have been shown to increase breastfeeding duration in areas with low breastfeeding rates [19]; however, parents in Vientiane did not agree with providing a cash incentive to support longer breastfeeding. Key informants responded positively as they felt it would give parent additional support. A study from the United Kingdom (UK) supports the opinions of key informant as financial incentives were shown to increase breastfeeding rates but suggested tailoring incentives to specific groups of women. Moreover, research among Women, Infants, and Children (WIC)–enrolled Puerto Rican mothers showed that financial incentives significantly increased breastfeeding rates and duration [20].

Parents recommended social media advertising and health education as supportive measures for breastfeeding instead of cash transfers. In the UK, the significant rise of Breastfeeding Support Facebook (BSF) groups has proven highly valuable, connecting mothers with local services and providing expertise and shared experiences conveniently and timely, thereby enhancing confidence and self-efficacy [21].

Our study also found that mothers request more attention from health providers in the postnatal period. Previous research has shown that engagement with health professionals for breastfeeding support is crucial [22]. Lactation clinics services and facilities to support breastfeeding, such as consultations, rooms, equipment, and specialists, have been shown to provide comprehensive lactation support and increases infant feeding practices [23].

Furthermore, the prevalence of EBF in Lao has shown an upward trajectory over time. In the 2000, 2006, 2011/2012, and 2017 surveys, the estimated exclusive breastfeeding rates were approximately 19.03%, 26.87%, 40.67%, and 44.89%, respectively. and several factors are significantly associated with EBF such as Region of Residence, Ethnicity, Wealth Index, and Child’s Age [24].

### Limitation of the study

Our study has limitations that should be considered. The sample size is limited to four districts in Vientiane province, making it non-representative of the entire population in the Lao People’s Democratic Republic. The findings cannot be generalized to all mothers, fathers, and key informants with children in the country. The study predominantly includes the Lao Loum ethnic group, potentially overlooking the diverse perspectives of other ethnic groups in the region. Respondents’ subjectivity may introduce discrepancies in the data. In addition, social desirability bias may have influenced participants’ responses, particularly given the inclusion of healthcare workers, community leaders, and elders as participants. The COVID-19 pandemic affected fieldwork, particularly in organizing focus group discussions.

## Conclusion

Our findings revealed that while many participants expressed support for exclusive breastfeeding for the first six months, they encountered significant barriers in practice. The key themes that emerged encompassed inadequate support from healthcare providers, challenges related to milk supply, employment status, maternal health, mode of delivery, and the perceived convenience of formula feeding.

In response to these identified challenges, we propose potential interventions tailored for mothers in Vientiane, Lao PDR. These interventions may include carefully designed cash transfer programs, particularly from a policy perspective, alongside non-monetary incentives, enhanced health education initiatives, and breastfeeding-supportive policies from the Ministry of Health. Strengthening the capacity of healthcare providers, leveraging social media and advertising for widespread awareness, and creating a supportive environment for breastfeeding are additional measures that could be impactful. Importantly, these findings provide formative qualitative evidence to inform the design and refinement of context-appropriate breastfeeding interventions in urban Lao settings.

Future research should focus on evaluating the feasibility, acceptability, and effectiveness of these interventions, particularly through implementation studies. It is our hope that this study serves as a foundational step toward the development of new intervention studies aimed at promoting breastfeeding in Vientiane, Lao PDR. By addressing these multifaceted challenges and implementing targeted interventions, this research contributes to improving breastfeeding practices and maternal and infant health in the region.

## Supplementary Information

Additional file 1: Interview guides used in the study (DOCX).This file contains the semi-structured interview guides used for focus group discussions and key informant interviews with mothers, fathers, healthcare workers, and community stakeholders.

## Data Availability

The data described in this manuscript is freely and openly accessible and is available on Google Drive at the following URL: https://drive.google.com/drive/folders/1 × 2Al_QZYGTeecf0TPNwcQLcgT5WF0v3e? usp=sharing. To ensure clarity and consistency in data collection, an interview guide was developed. The English language version of the interview guide can be found in the supplementary materials (Additional file 1) **.**.

## Abbreviations

A&T: Alive & Thrive
CDN: Current developments in nutrition
CDHS: Cambodian demographic and health survey
ECBF: Exclusive breastfeeding
FGD: Focus group discussion
IDI: In-depth interview
Lao PDR: Lao People’s Democratic Republic
PW: Pregnant women
VITERBI: Vientiane multi-generational birth cohort
UNICEF: United Nations International Children’s Fund
USA: United States of America
NPAN: National plan of action for nutrition
WIC: Women, infants, and children
WHO: World Health Organization
WHA: World Health Assembly
