# Supplementary material for: “People who have money feed formula to their infants”: a qualitative study of exclusive breastfeeding barriers and potential interventions in Lao People’s Democratic Republic
Source: BMC Public Health. 2026 Apr 23;26:1827. doi: 10.1186/s12889-026-27416-y (PMC13248451; doi:10.1186/s12889-026-27416-y)
Supplement: Supplementary file 1 — Additional file 1: Interview guides used in the study (DOCX).This file contains the semi-structured interview guides used for focus group discussions and key informant interviews with mothers, fathers, healthcare workers, and community stakeholders. [file 12889_2026_27416_MOESM1_ESM.zip › 5 VITERBI CRF Key Informant Interview Guide-28 Sept 2020.docx]

**Key Informants (elders/village leaders; Ministry of Health Staff)**

1. Can you tell me a little bit about the context of breastfeeding in Laos?
   1. What is the history of infant feeding practices here? (Possible prompts: how have women traditionally fed their infants, has that changed over time? Why)
   2. (For those in policy) What efforts have there been on the policy level to support/encourage breastfeeding (i.e. baby friendly hospitals etc.)?
   3. Is breastfeeding in Laos culturally acceptable? Why or why not?
      1. Do women breastfeed in public? Around family members? Around friends?
2. If you were to design a program that encouraged women to breastfeed – what would that look like?
   1. Would you focus on policy, organizational change, or supporting the family directly?
   2. What type of program do you think would have the most immediate change? The most long-term, sustainable change?
3. Now I’d like to ask you about programs to support breastfeeding focus on providing direct incentives to the mother, such as receiving monthly payments as long as she is breastfeeding. Do you think this would encourage breastfeeding?
   1. Can you think of any reasons why this might be problematic?
   2. Besides money, what other types of direct incentives (gifts such as books or toys, or services) might help a woman start breastfeeding or breastfeed longer?

**Elders/Village Leaders Only**

1. What are the infant feeding practices you recommend? Why?
2. Who has a role in deciding how a woman feeds her infant?
   1. What role do you feel like you have in shaping infant feeding practices as an elder/village leader?
   2. Do you support women’s decision to breastfeed or not breastfeed?
3. Now I’d like to ask you a very open-ended question, and I want to encourage you to share whatever comest to mind - what would you like to see happen related to infant feeding in your community?

**Closing**

1. Do you have any further comments or thoughts on breastfeeding you would like to share with us, that I haven’t asked?
