# Supplementary material for: “People who have money feed formula to their infants”: a qualitative study of exclusive breastfeeding barriers and potential interventions in Lao People’s Democratic Republic
Source: BMC Public Health. 2026 Apr 23;26:1827. doi: 10.1186/s12889-026-27416-y (PMC13248451; doi:10.1186/s12889-026-27416-y)
Supplement: Supplementary file 1 — Additional file 1: Interview guides used in the study (DOCX).This file contains the semi-structured interview guides used for focus group discussions and key informant interviews with mothers, fathers, healthcare workers, and community stakeholders. [file 12889_2026_27416_MOESM1_ESM.zip › 5 VITERBI CRF Guide_Health Care Workers-28 Sept 2020.docx]

Interviewer Initials |__|__|__| Date |__|__/__|__/__|__|

**Introduction**

Hello, my name is _____. I am from the Lao Tropical and Public Health Institute working on a project about infant health. I would like to ask you some questions to try and understand your opinion on things related to your child/ren. This information will be used to inform a program focused on supporting breastfeeding mothers and encouraging child development. We really appreciate that you take the time for this interview. Your inputs, thoughts and opinions will be very valuable to understand you and your community. Do you have any questions before we start?

*Fill out consent form< which will include a component about audio recordings>

**General Infant Feeding Questions**

I’d like to ask you a few questions about the women you see in your [insert medical setting]and how they make decisions about how they’ll feed their baby.

1. To start, can you tell me a little bit about how women in your clinic/practice/medical center choose to feed their infants.
   1. In your experience who makes that decision?
   2. What role does the baby’s father have in that decision?
   3. What are the factors that guide that decision (e.g. health of the infant, convenience, support of the family, etc.)?
   4. What is the general practice of the parents/relatives regarding the first food to be given to the babies? Why?
2. Do you talk to patients while they’re pregnant, about how they plan to feed their babies?
   1. (If so): What do you recommend? Why?

**Breastfeeding Questions**

1. What do you see as your role (if any) in educating or supporting women to breastfeed?
2. What do you think is important for all pregnant women to know about breastfeeding?
3. Why do you think so many women stop breastfeeding within a few weeks or months after giving birth?

**Incentives for Breastfeeding**

1. If you were to design a program that encouraged women to breastfeed once the baby was born – what would that look like?
   1. Would you focus on supporting the family directly (e.g. the pregnant women, their mothers/families, or the baby’s father) or programs implemented within healthcare settings?
   2. Do you think this would create sustainable change? If not, what recommendations do you have for supporting breastfeeding among more women that lasts beyond a specific program?
2. Some programs to support breastfeeding focus on providing direct incentives to the mother, such as receiving monthly payments as long as she is breastfeeding. Do you think this would encourage breastfeeding?
   1. Can you think of any reasons why this might be problematic?
   2. Besides money, what other types of direct incentives (gifts such as books or toys, or services) might help a woman start breastfeeding or breastfeed longer?

**Closing**

1. Do you have any further comments or thoughts on breastfeeding you would like to share with us, that I haven’t asked?
