# Supplementary material for: “People who have money feed formula to their infants”: a qualitative study of exclusive breastfeeding barriers and potential interventions in Lao People’s Democratic Republic
Source: BMC Public Health. 2026 Apr 23;26:1827. doi: 10.1186/s12889-026-27416-y (PMC13248451; doi:10.1186/s12889-026-27416-y)
Supplement: Supplementary file 1 — Additional file 1: Interview guides used in the study (DOCX).This file contains the semi-structured interview guides used for focus group discussions and key informant interviews with mothers, fathers, healthcare workers, and community stakeholders. [file 12889_2026_27416_MOESM1_ESM.zip › 5 VITERBI CRF Focus Group Guide_Fathers-28 Sept 2020.docx]

Interviewer Initials |__|__|__| Date |__|__/__|__/__|__|

**Introduction**

Hello, my name is _____. I am from the Lao Tropical and Public Health Institute working on a project about infant health. I would like to ask you some questions to try and understand your opinion on things related to your child/ren. This information will be used to inform a program focused on supporting breastfeeding mothers and encouraging child development. We really appreciate that you take the time for this interview. Your inputs, thoughts and opinions will be very valuable to understand you and your community. Do you have any questions before we start?

*Fill out consent form< which will include a component about audio recordings>

**Introduction**

I would like us to do a round of introductions to help us get to know each other a little better.

1. Can you start by providing your name and age, and number of kids and their ages.

**General breastfeeding questions**

1. Can you tell me a little bit about what you know about breastfeeding?
   1. Can be positive (e.g. health benefits etc.) or negative (makes it harder to leave the baby with others, woman’s time is occupied with baby)
2. How do you feel about breastfeeding? (Actively support/encourage both positive and negative responses)
   1. What has shaped your feelings about breastfeeding?
   2. Has that changed since having a baby (e.g. did you feel differently before)?

**Infant Feeding Experiences**

1. When your wife/partner was pregnant, how was the decision about how to feed the baby made?
2. What or whom informed that decision?
   1. Prompt (if no one mentioned their role): Did you ever talk with your wife/partner about breastfeeding? What did you discuss?
   2. Did you support your partners decision to breastfeed or not breastfeed? If she went against your wishes, how did you feel?
3. Would you be willing to talk to your wife about breastfeeding if you hear something about it and felt she did not know it? If yes, proble.

**Incentives for Breastfeeding**

1. If you were to design a program that encouraged women to breastfeed once the baby was born – what would that look like?
   1. Should the program focus more on women, or the men? Why?
2. One option is to give women a small gift (e.g. books or toys for her child), or a small amount of money while she is breastfeeding, what is your opinion of that?
   1. Can you think of any reasons why this type of program might be problematic?
   2. Do you think such programs make sense? Do you think they are fair?
   3. How do you think men in Laos would respond if women received money for breastfeeding?
   4. What things (other than money) would be useful for a family to receive, to support/encourage breastfeeding?

**Decision Making**

1. In your opinion, is it acceptable for a woman to breastfeed around her husband? in public? Around family members? Around friends?
2. In your household, who decides how to use cash and how is this decision made? Why? If you disagree on these decisions how is the disagreement resolved?
3. Do you think it is appropriate for women to earn and manage household money? Please explain.
4. In your household, who decides when to send children to school? Are decisions different when they are made regarding boys’ and girls’ education? Why?

**Closing**

1. Do you have any further comments or thoughts on breastfeeding you would like to share with us, that I haven’t asked?
