# Supplementary material for: “People who have money feed formula to their infants”: a qualitative study of exclusive breastfeeding barriers and potential interventions in Lao People’s Democratic Republic
Source: BMC Public Health. 2026 Apr 23;26:1827. doi: 10.1186/s12889-026-27416-y (PMC13248451; doi:10.1186/s12889-026-27416-y)
Supplement: Supplementary file 1 — Additional file 1: Interview guides used in the study (DOCX).This file contains the semi-structured interview guides used for focus group discussions and key informant interviews with mothers, fathers, healthcare workers, and community stakeholders. [file 12889_2026_27416_MOESM1_ESM.zip › 5 VITERBI CRF Focus Group Guide_Mothers-28 Sept 2020.docx]

Interviewer Initials |__|__|__| Date |__|__/__|__/__|__|

**Introduction**

Hello, my name is _____. I am from the Lao Tropical and Public Health Institute working on a project about infant health. I would like to ask you some questions to try and understand your opinion on things related to your child/ren. This information will be used to inform a program focused on supporting breastfeeding mothers and encouraging child development. We really appreciate that you take the time for this interview. Your inputs, thoughts and opinions will be very valuable to understand you and your community. Do you have any questions before we start?

*Fill out consent form< which will include a component about audio recordings>

**Introduction**

I would like us to do a round of introductions to help us get to know each other a little better.

1. What is the best part of your week so far?
2. Can you tell me your name and age, and number of kids and their ages.

**General Breastfeeding Questions**

I’m going to start out by asking you some general questions about women in Laos, such as their decisions and experiences about how to feed their babies. I’ll be asking questions later about your own thoughts and experiences, but for now we’ll start more generally.

1. In your opinion, what do most women in your community think of breastfeeding?
   1. Possible prompts (Use these only if necessary): Beautiful and natural, or ugly and unpleasant? Convenient or a hassle? Difficult or easy?
   2. What do you think shapes that? (Possible prompts: where a woman is from or where she currently lives, or her education level?)
2. Can you tell me a little bit about how most pregnant women in your community initially plan on feeding their babies?
   1. Why do you think they choose that?
      1. Possible prompt: Who do you think has the biggest influence on whether a woman breastfeeds (e.g. medical providers, partner, spouse, friends, family, etc.)?
      2. What are women told by midwives or birth attendants about how to feed their baby?
3. Do you think some women plan on not breastfeeding their child?
   1. Why do you think they choose that?
4. Now, can you tell me a little about how most women feed their babies once they’re born?
   1. What do women typically do with colostrum (first milk after birth)?
   2. I understand some women give their baby water or other milks before their own milk comes in, can you tell me a little about that?
   3. Why do you think some women don’t breastfeed?
   4. What about as they get older? How old is the baby once the women typically stops breastfeeding? Why do they stop?
   5. How do you think breastfeeding affects the health of the baby?
5. In your opinion, what do most men in your community think of breastfeeding?
   1. Why might some men want the woman to breastfeed?
   2. Why might they not want the woman to breastfeed?
6. Who would you ask if you needed advice about breastfeeding?
   1. What would happen if you didn’t follow their advice?
7. In some cultures, women may breastfed a child that is not theirs, what do you think of this practice?

**Infant Feeding Experiences**

Now I’m wondering if you could tell me a little bit about how you fed your infant(s). Remember there’s no wrong answer here, we’re just trying to understand what it was like for you.

1. When your most recent baby was born, did you ever breastfeed?
   1. Why or why not?
2. How did your partner/husband feel about breastfeeding?
3. At what age did you start giving the child other liquids? What did you give them and why?
4. At what age did you start to give the child porridge or other easy to digest food? What did you give them?
5. At what age of the child did you stop breastfeeding altogether? Why did you stop breastfeeding?

**Incentives for Breastfeeding**

1. If you were to design a program that encouraged women to breastfeed once the baby was born – what would that look like?
2. One option is to give women a small gift (e.g. books or toys for her child), or a small amount of money while she is breastfeeding, what is your opinion of that?
   1. Do you think such programs make sense? Do you think they are fair?
   2. How do you think the partner/husband would feel about that?
   3. (If they think money is a good idea) How much money do you think should be given?
   4. Can you think of any reasons why this type of program might be problematic?
   5. Are there any other things you could think of that might help a woman start breastfeeding or breastfeed longer?

**Decision Making**

1. In your household, who decides how to use cash and how is this decision made? Why? If you disagree on these decisions how is the disagreement resolved?
2. Do you think it is appropriate for women to earn and manage household money? Please explain.
3. In your household, who decides when to send children to school? Are decisions different when they are made regarding boys’ and girls’ education? Why?

**Closing**

1. Do you have any further comments or thoughts on breastfeeding you would like to share with us, that I haven’t asked?

Once again thank you very much for your participation.
